# Making new connections: an fNIRS machine learning classification study of inter-brain synchrony in the default mode network

**DOI:** 10.1093/scan/nsag044

**Published:** 2026-07-22

**Authors:** Grace Qiyuan Miao, Ian J Lieberman, Ashley L Binnquist, Agnieszka Pluta, Bear M Goldstein, Rick Dale, Matthew D Lieberman

**Affiliations:** Department of Communication, University of California, Los Angeles, CA 90095, United States; Department of Data Science, Stanford University, Stanford, CA 94305, United States; Department of Psychology, University of California, Los Angeles, CA 90095, United States; Faculty of Psychology, University of Warsaw, Warsaw, 02-097, Poland; Department of Psychology, University of California, Los Angeles, CA 90095, United States; Department of Communication, University of California, Los Angeles, CA 90095, United States; Department of Psychology, University of California, Los Angeles, CA 90095, United States

**Keywords:** functional near-infrared spectroscopy (fNIRS), inter-brain synchrony, default mode network, social connection, communication

## Abstract

Little is known about the neurocognitive underpinnings of forming social connections. In this study, we tested whether default mode network (DMN) inter-brain synchrony (which prior work has linked to interpersonal alignment, or “seeing eye-to-eye”) predicts feelings of connection during conversations. Seventy pairs of strangers engaged in shallow or deep conversations while brain activity was measured with functional near-infrared spectroscopy (fNIRS). Stranger dyads in the deep condition felt more connected than those in the shallow condition. Greater feelings of connection were associated with increased DMN synchrony, with the right temporoparietal junction (TPJ) subregion of the DMN emerging as the strongest individual predictor across regression analyses and machine learning classification. These findings suggest that DMN synchrony in general and TPJ synchrony in particular may play a key role in strangers coming to “see eye-to-eye” during face-to-face interactions and feeling more socially connected.

## Introduction

The current loneliness epidemic has major negative consequences for public health and well-being (Murthy 2020; Park *et al*. 2020; Hong *et al*. 2023). Increasing social connections with others is crucial to address (Masi *et al.* 2011), yet little is known about the neurocognitive bases of connection formation.

Increasingly, neuroscientists use interpersonal “inter-brain synchrony” within the default mode network (DMN) as a measure of when two or more people subjectively experience something in similar ways (i.e. “seeing eye-to-eye”; Lieberman 2025). The decision to focus on the DMN in this context is motivated by its hypothesized role as a “sense-making” network, integrating extrinsic information with prior knowledge to construct rich, context-dependent internal narratives (Mars *et al.* 2012; Meyer 2019; Yeshurun *et al.* 2021; Lieberman 2022; Menon 2023). Throughout this manuscript, we use the term “DMN inter-brain synchrony” to refer to between-person synchrony within DMN regions, or the temporal correlation of brain activity between two interacting individuals.

During passive viewing of naturalistic stimuli (e.g. movie clips), multiple functional magnetic resonance imaging (fMRI) studies have demonstrated that greater inter-brain synchrony within DMN regions is linked to both shared ways of experiencing (Yeshurun *et al.* 2017; Nguyen *et al.* 2019; Saalasti *et al.* 2019; Dieffenbach *et al.* 2021) and stronger real-world social connections, including friendship and marriage (Parkinson *et al.* 2018; Li *et al.* 2022). Additionally, a recent fMRI study by Speer *et al.* (2024) examined brain responses between friends and stranger dyads during conversations, finding that friends’ brain activity diverged in “neural state space,” while strangers’ brain activity converged. Their study revealed the potential role of convergence in dyadic interaction, though it did not focus on or reveal patterns that predicted feelings of connection.

Although functional near-infrared spectroscopy (fNIRS) has become a widely used method to study neural dynamics during dyadic conversations (Kelsen *et al.* 2022), no study to date has directly examined how inter-brain synchrony relates to the subjective experience of connection generated through real-time interaction.

To address this gap, the present study focuses exclusively on stranger dyads and examines differences in DMN synchrony between pairs who report feeling connected after the conversation and those who do not. This design allows us to isolate neural patterns specifically associated with successful connection-building and to explore the dynamic neural underpinnings of early-stage interpersonal alignment. Our goal was to examine whether DMN synchrony during active conversations predicts social connection, similar to findings in passive viewing studies (Parkinson *et al.* 2018; Li *et al.* 2022). If, as prior work suggests (Yeshurun *et al.* 2017; Nguyen *et al.* 2019; Saalasti *et al.* 2019; Dieffenbach *et al.* 2021; Lieberman 2025), DMN synchrony reflects two people seeing eye-to-eye, DMN synchrony during conversation ought to predict self-reported feelings of connection afterwards.

To examine this, we recruited 140 participants (forming 70 dyads of unacquainted individuals) for a face-to-face conversation task while measuring neural activity via fNIRS. Dyads were randomly assigned, in a between-subjects design, to converse using either shallow or deep prompts (Kardas *et al.* 2022), designed to elicit varying levels of interpersonal connection. Shallow prompts included topics such as the weather, haircuts, and TV shows, while deep prompts asked about participants’ perfect day, moments of vulnerability, or times they cried in front of others.

We hypothesized that DMN synchrony and conversation depth (experimentally manipulated and self-reported) would predict the degree of self-reported connection present between strangers. We tested these hypotheses using both regression analyses and machine learning-based classification analyses. As our fNIRS montage also captured data from other neural networks, including the frontoparietal network (FPN), the dorsal attention network (DAN), and the ventral attention network (VAN), we examined each of these as well, but had no *a priori* hypotheses for these networks.

## Materials and Methods

### Participants

A total of 210 individuals participated, forming 105 stranger dyads. All 105 dyads (23 male-male, 38 female-female, 39 male-female, 3 female-other, and 2 male-other) contributed to behavioral analyses. A subset of 70 dyads (20 male-male, 24 female-female, 23 male-female, 2 male-other, and 1 female-other) additionally contributed to neuroimaging analyses. The reduction from 105 to 70 dyads reflects a hardware issue with the fNIRS system that was identified after the first 35 dyads had been run; data from those 35 dyads were retained for behavioral analyses but excluded from the neuroimaging analyses. Behavioral patterns observed in the 70-dyad neuroimaging subsample were consistent with those in the full 105-dyad sample.

Participants were recruited from the UCLA Departments of Psychology and Communication subject pools as well as flyers on the UCLA campus. Experimental procedures were approved by the UCLA Institutional Review Boards (#22-001209), and participants provided informed consent prior to the start of the study. Participants received either two course credits or a $30 gift card for the 2-hour-long experiment. All participants self-reported as having English as their primary language.

### Experimental procedures

This was a between-subjects experimental design with two conditions: shallow and deep (among 105 dyads for behavioral analysis—shallow condition: 12 male-male, 20 female-female, 19 male-female, and 2 male-other; deep condition: 11 male-male, 18 female-female, 20 male-female, and 1 female-other; among 70 dyads for neuroimaging analysis—shallow condition: 10 male-male, 13 female-female, 11 male-female, and 2 male-other; deep condition: 10 male-male, 11 female-female, 12 male-female, and 1 female-other).

**Figure 1:**
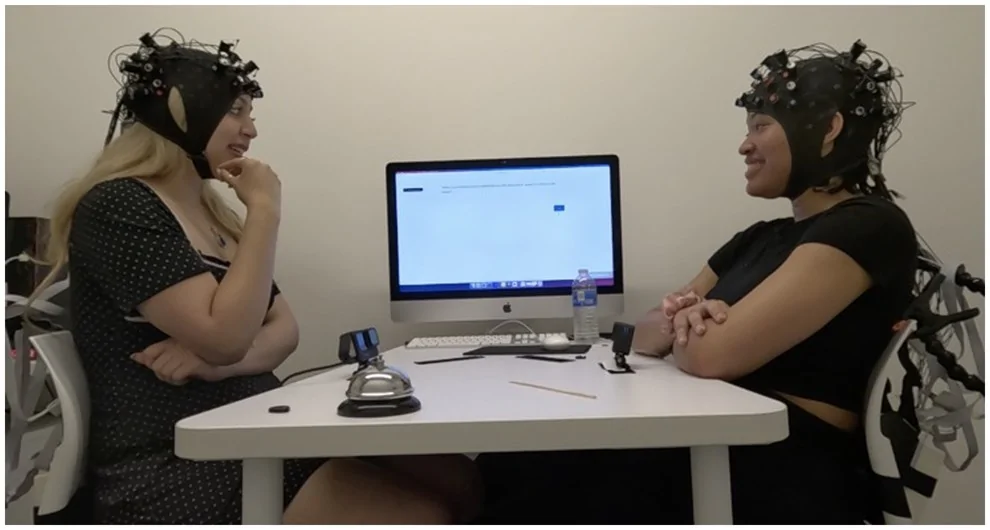
Experiment setup. Stranger dyads are equipped with fNIRS. Three GoPro cameras are placed in the room—two capturing the two participants’ facial expressions, and one capturing the scene from a third-person perspective.

In each session, two strangers engaged in a face-to-face, get-to-know-you conversation, with either shallow or deep topics displayed sequentially on a computer screen (Fig. 1). During the experiment, participants wore an fNIRS rig. Three GoPro cameras were placed in the room to record conversations and nonverbal behaviors. One camera was positioned in front of each participant to capture facial expressions, and a third camera captured both participants together. Participants also completed questionnaires regarding their personal traits and experiences with the conversation.

The conversation topics (Table 1) were adapted from Kardas *et al.* (2022). These topics appear one by one in the same order on a computer screen accessible to both participants. Participants read the question at the same time and volunteered to answer in the order they preferred. Participants were instructed to stay on topic, take however much time they needed for every prompt, and when they finished discussing one topic, click a button to move on to the next. Each session was designed to last 20 min and took place without the presence of experimenters, allowing for a more natural conversation flow.

**Table 1:** Conversation topics assigned to participants.

Conversation topics	
Shallow Condition	1. What do you think about the weather today? 2. How often do you come to UCLA campus? 3. How did you celebrate last Halloween? 4. How often do you get your hair cut? Where do you go? Have you ever had a really bad haircut experience? 5. What is the best TV show you’ve seen in the last month? Tell your partner about it. 6. When was the last time you walked for more than an hour? Describe where you went and what you saw. 7. Do you like to get up early or stay up late? Why? 8. Do you have anything planned for later today? When are you going to do it? 9. Can you describe a conversation you had with another person earlier today? 10. What’s your daily routine like? 11. What’s the most stupid thing you have seen on TikTok? 12. What’s your favorite exercise? 13. What’s the last movie you saw in the theater? 14. What’s your favorite social media? 15. What’s your favorite meme? 16. What are your three favorite songs at the moment? 17. What restaurants do you recommend for friends from out of town? 18. Do you like hats? What kind? 19. What’s your sign in astrology? 20. Do you prefer the beach or the mountain? 21. You have answered all the questions the experimenter provides. Feel free to talk about anything you want until the time is up!
Deep Condition	1. What would constitute a “perfect” day for you? 2. Where is somewhere you’ve visited that you felt really had an impact on who you are today? 3. If you were going to become a close friend with the other participant, please share what would be important for him or her to know. 4. If a crystal ball could tell you the truth about yourself, your life, the future, or anything else, what would you want to know? 5. For what in your life do you feel most grateful? Tell the other participant about it. 6. Is there something you’ve dreamed of doing for a long time? Why haven’t you done it? 7. What is one of the more embarrassing moments in your life? 8. What is one of your most meaningful memories? Why is it meaningful for you? 9. Can you describe a time you cried in front of another person? 10. If you could undo one mistake you have made in your life, what would it be and why would you undo it? 11. You have answered all the questions the experimenter provides. Feel free to talk about anything you want until the time is up!

Note: Questions 1–10 from both the shallow and deep conditions have been adapted from Kardas *et al.* (2022). Additional questions were added to the shallow condition for sufficient content in a 20-min discussion.

### fNIRS data acquisition and preprocessing

**Figure 2:**
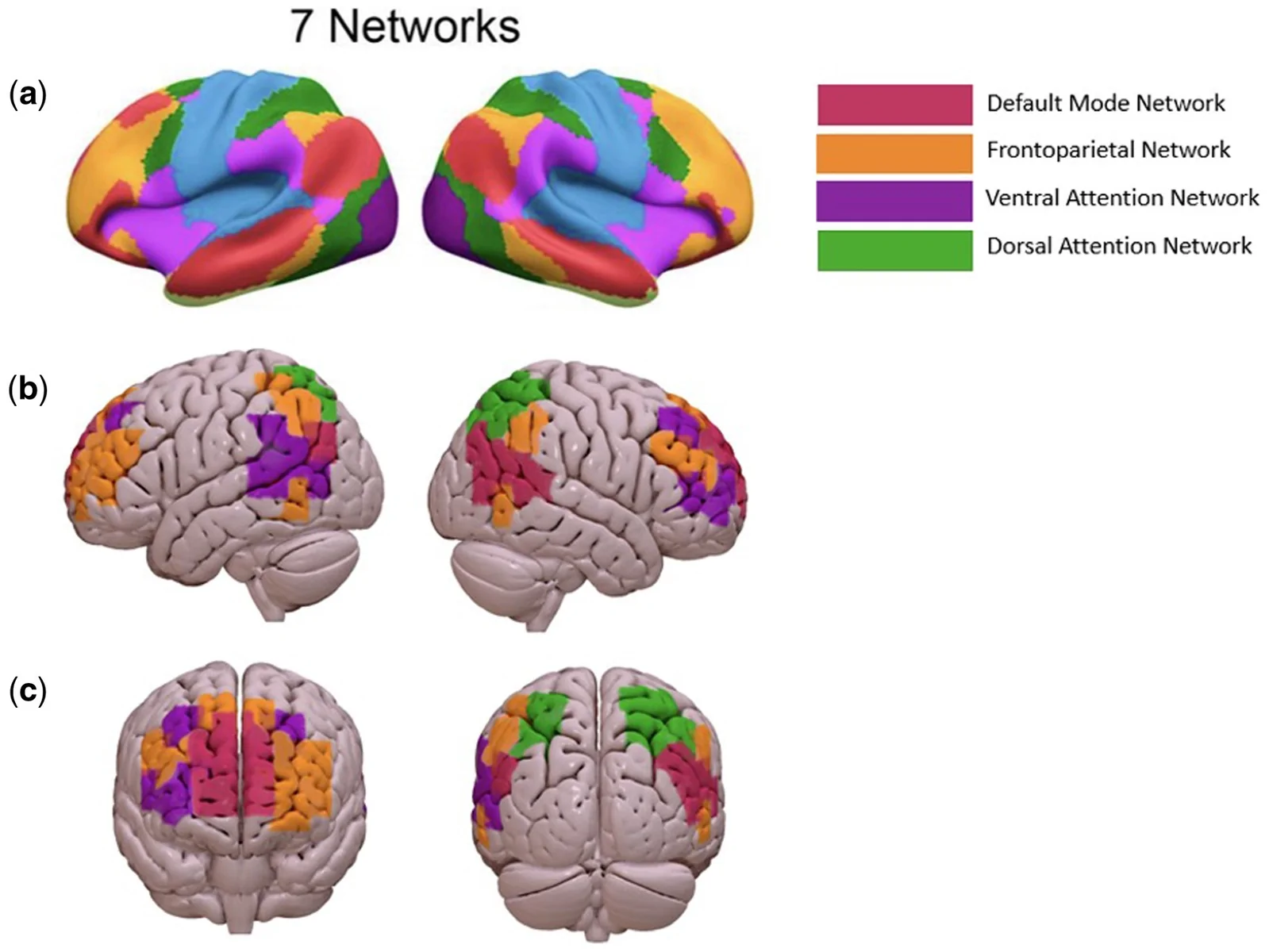
fNIRS montage consists of 40 channels for partial-brain coverage of cortical regions implicated in social interactions, spanning four networks: the default mode network (DMN), the frontoparietal network (FPN), the ventral attention network (VAN), and the dorsal attention network (DAN). (a) Seven-network cortical parcellation defined by Yeo *et al*. (2011). (b) Lateral views of DMN, FPN, VAN, and DAN. (c) Anterior (left) and posterior (right) views of the same four networks.

Participants were scanned using a mobile fNIRS system (NIRSport2 by NIRx Medical Technologies, LLC, NY). The probe layout was comprised of 16 light sources and 16 detectors with a 3-cm average source-detector separation distance, which forms 40 channels (source-detector pairs) for partial-brain coverage across mentalizing (i.e. medial prefrontal cortex (mPFC) and temporoparietal junction (TPJ)) and working memory regions (i.e. lateral prefrontal cortex (lPFC) and superior parietal lobule (SPL)) (Fig. 2). The montage layout was created in accordance with the 10-10 UI external positioning system to ensure consistency across head sizes. We measured participants’ head sizes and then fitted them with caps of appropriate sizes that affix the optodes to the scalp. Raw light intensity data were collected at a sampling rate of 5.09 Hz at wavelengths of 760 and 850 nm.

Collected NIRS data underwent a comprehensive preprocessing pipeline. This pipeline utilized custom scripts in MATLAB alongside the Homer2 software suite (Huppert *et al.* 2009), adhering to established fNIRS best practices (Yücel *et al.* 2021). Emphasis was placed on analyzing oxyhemoglobin (HbO) concentrations, which prior research has indicated are more responsive to changes in cerebral blood flow than deoxyhemoglobin (HbR) levels (Pan *et al.* 2017).

The preprocessing began with removing unrelated data: each time course was truncated based on a trigger that indicated the start of the conversation. Noisy and oversaturated channels were identified and excluded using a modified quartile coefficient of dispersion (Bonett 2006), with specific thresholds adjusted for the sampling rate (Cthresh = 0.6-0.03*sampling rate). Further refinement of the data included corrections for motion and non-neural changes in blood oxygenation. To address motion artifacts, discrete wavelet transform techniques (Molavi and Dumont 2012) were performed to remove spike artifacts. To address non-neural physiological influences (e.g. cardiac and respiratory rhythms) and baseline drift, a conservative bandpass filter (0.008-0.2 Hz) was applied. Past work suggests that the cognitive dynamics of interest in this study are primarily manifested in lower frequency ranges (Zuo *et al.* 2010; Sasai *et al.* 2011).

Filtered data were then transformed from optical density to hemoglobin concentration values. This conversion used the modified Beer-Lambert Law (MBLL) with a standard differential path length factor [6, 6], commonly applied to adult cortical tissue to account for light dispersion. The final quality control step involved an autocorrelation change assessment to gauge the impact of motion correction. Channels displaying a substantial change in autocorrelation (exceeding a threshold of *r* = 0.1) were deemed significantly influenced by motion and thus excluded from subsequent analyses.

### Behavioral analyses for conversation and connection

Participants completed a post-conversation survey using a 5-point Likert scale, rating their experience in terms of bonding, engagement, liking, enjoyment, interest, awkwardness, ease, and perceived depth of the conversation; as well as epistemic knowledge, and similarity to the partner; and willingness for future interaction and the potential to become friends in real life.

We created the “connection” composite variable by averaging the measures from three items (bonding, engagement, and liking) with the greatest correlation between themselves (*r *> 0.6). We then calculated the mean within-dyad connection, which is used in both behavioral and neural analysis below.

To induce variability of connection across stranger dyads, we introduced shallow and deep topics of conversation as experimental conditions. However, with the naturalistic nature of conversations, we expected dyads to show variability in conversation depth not fully captured by condition. Thus, we report behavioral findings based on condition assignment as well as on self-reported felt depth of the conversation and use each as predictors of connection.

### Inter-brain synchrony across brain networks and anatomical ROIs

Yeo *et al.* (2011) identified a 7-network parcellation of the human cerebral cortex based on 1000 subjects. These networks converged and extended on networks previously described in resting-state literature, including the DMN, the FPN, the VAN, and the DAN (Fig. 2). We first divided the cortical areas covered by the 40 channels in our fNIRS montage based on the Yeo parcellation. We then obtained inter-brain synchrony scores by computing Pearson’s correlation for the average time series for each participant within each of the networks.

Since our main hypothesis focused on DMN, we also analyzed sub-regions within the DMN (namely, medial PFC, left TPJ, and right TPJ) to further understand the brain responses. For these analyses, we strictly separated lateralized ROIs and disregarded two channels on the border of medial PFC and lateral PFC.

### Predicting felt connection by inter-brain synchrony in DMN

**Figure 3:**
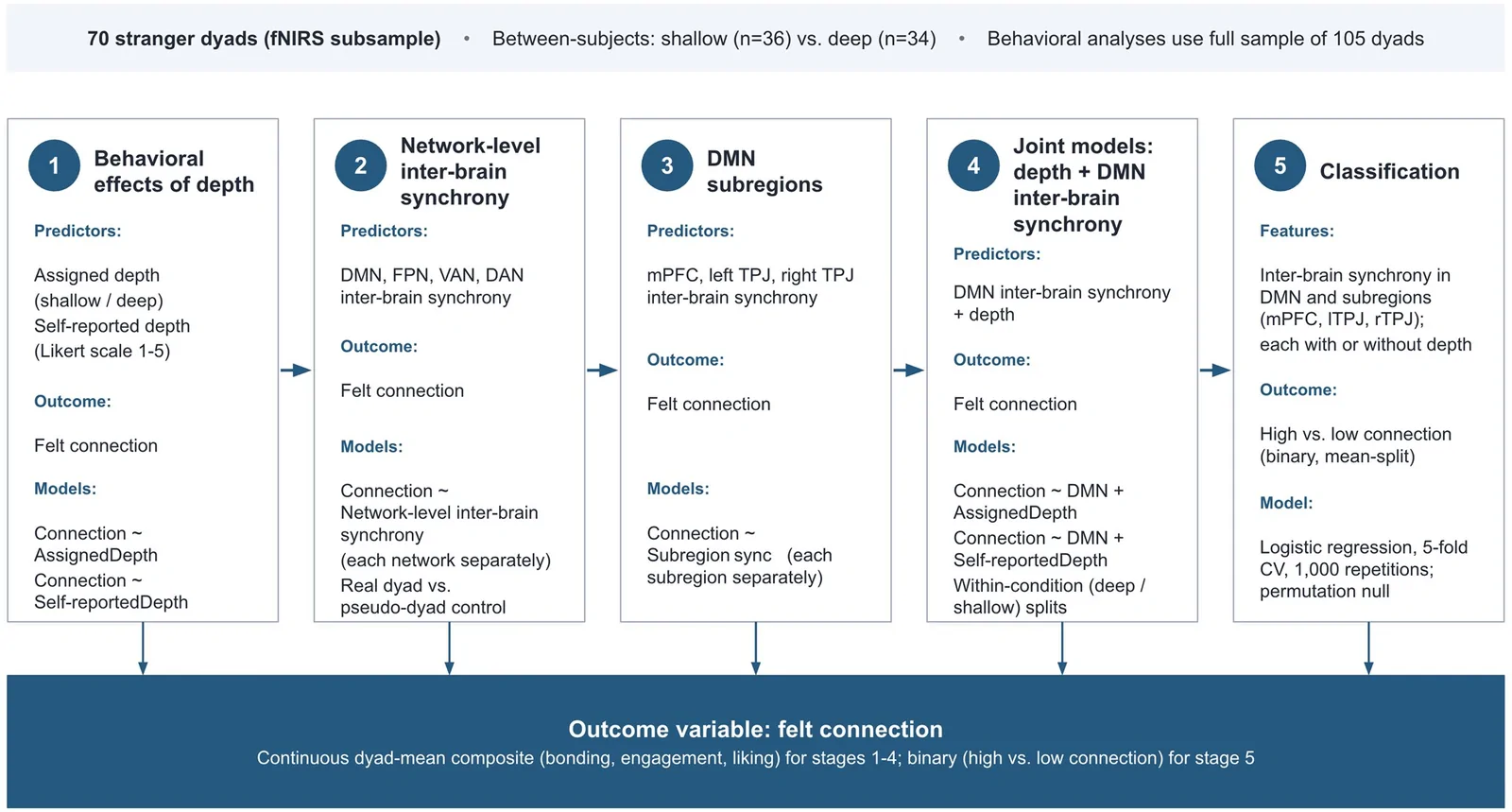
Analysis pipeline. Five sequential analytic stages relating conversational depth and dyadic inter-brain synchrony to felt connection between strangers. Stages 1-4 use linear regression to predict the continuous connection composite; stage 5 reframes the question as binary classification (high versus low connection) using logistic regression with five-fold cross-validation.

Figure 3 provides a summary of our analysis pipeline. We used linear regression to examine the extent to which the four neural networks (DMN, FPN, VAN, DAN) predicted connection, and then examined which lateralized DMN ROIs predicted connection. We then integrated the neural findings with the behavioral findings related to felt depth by conducting a linear regression analysis with DMN inter-brain synchrony and condition assignment, as well as DMN inter-brain synchrony and felt depth as predictors of connection.

To examine whether inter-brain synchrony effects in the real dyads could be explained by spurious alignment unrelated to actual interaction, we created pseudo-dyads by randomly pairing participants within the same condition who did not converse with one another in the experiment. This analysis provides a control for the possibility that synchrony may arise epiphenomenally from parallel processing of similar external inputs rather than genuine interpersonal coupling (Carollo *et al.* 2025; Atias *et al.* 2026). Pseudo-dyad synchrony scores were computed using the same preprocessing and network-level analytic pipeline as the real dyads and were used as a comparison baseline in subsequent analyses.

We also used a machine learning approach with binary classification, implemented using MATLAB’s fitclinear function (MATLAB (Version 2022b) [computer software] 2022), to examine whether high and low connection dyads could be predicted based on network synchrony and felt depth. All 70 dyads were categorized into high (*N* = 35) and low (*N* = 35) connection groups based on their average connection scores, with scores above 3.33 classified as high and those below 3.17 as low. A five-fold cross-validation strategy was employed to mitigate overfitting, wherein the dataset was randomly partitioned into approximately five equal folds. For each iteration, four folds were used for training, which comprised 80% of the dataset, while the remaining fold, which was 20% of the dataset, served as the test set. To enhance the robustness of classification performance estimates, the entire cross-validation procedure was repeated 1,000 times with different random partitions. Model performance was quantified by mean classification accuracy, averaged across all repetitions and cross-validation folds, with standard deviation computed to assess variability. To determine if the model was significant, the mean accuracy of the predictive model was compared to a null distribution, which was created by randomly shuffling the outcome of the connection for 1,000 repetitions.

## Results

### Behavioral results

**Figure 4:**
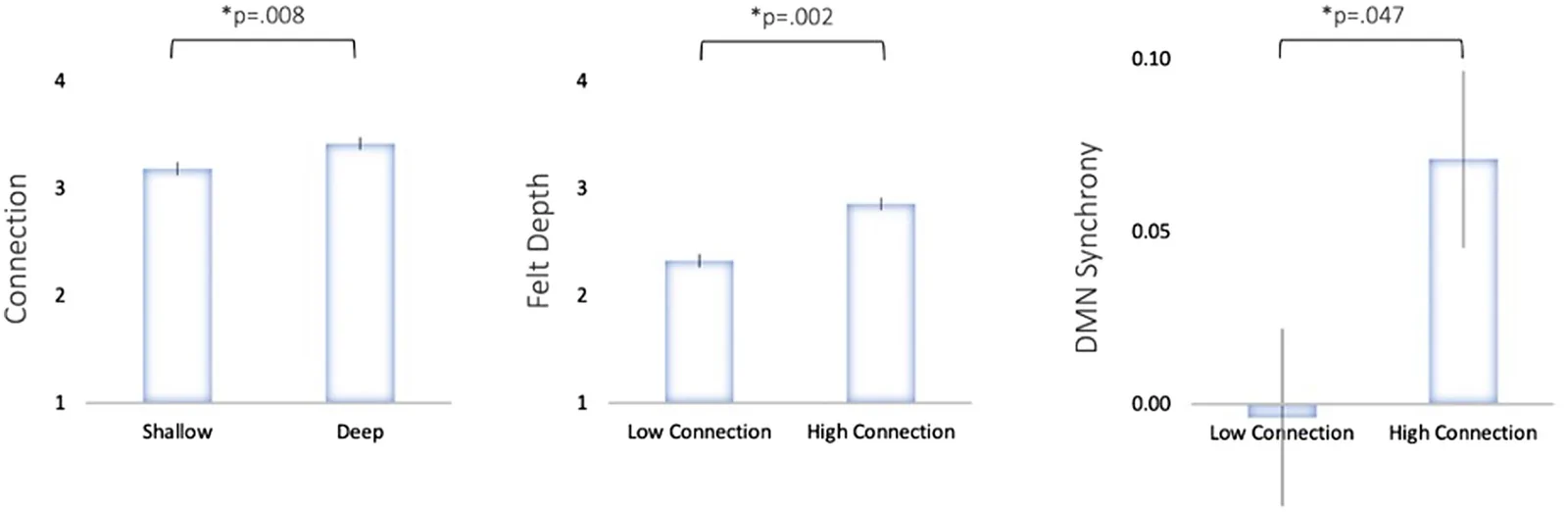
Difference across groups in behavioral and neural signals. Left: Deep conversation dyads reported significantly greater felt connection after conversation than shallow conversation dyads. Middle: High connection dyads reported greater felt depth of the conversation than low connection dyads. Right: High connection dyads generated greater neural synchrony in the DMN during conversation than low connection dyads.

Overall, participants felt moderately connected to each other after the conversation. The average self-reported connection for the full sample (*M* = 3.30) was significantly higher than the composite connection scale midpoint (*t*(104) = 6.62, *P* < .0001). Replicating Kardas *et al.* (2022), those in the deep conversation condition (*M* = 3.42) felt more connected than those in the shallow conversation condition (*M* = 3.18) (*t*(103) = 2.71, *P* = .008) (Fig. 4 left).

These same general trends held true for the 70 dyads whose fNIRS data were collected. Self-reported connection was significantly higher (*M* = 3.30) than the composite midpoint (*t*(68) = 5.44, *P* < .0001), and those in the deep condition (*M* = 3.41) felt marginally more connected than those in the shallow condition (*M* = 3.20) (*t*(68) = 1.96, *P* = .055). All additional analyses will focus on these 70 dyads with fNIRS data.

To account for the natural and unpredictable nature of human conversation, we analyzed both the assigned shallow/deep condition and participants’ self-reported experience of conversation depth. Although participants were randomly assigned to shallow or deep conditions, conversational trajectories sometimes diverged from these assignments. For example, a shallow topic about the weather could transition into a deep discussion about childhood because the weather that day reminds them of their hometown. Alternatively, a deep topic about embarrassing moments may not actually deepen the conversation if one of the participants is unwilling to share about themselves.

Indeed, 28.6% of dyads (20 of 70) reported self-perceived depth that was inconsistent with their assigned condition. Specifically, 8 of the 36 dyads in the shallow condition reported higher-than-average conversation depth, whereas 12 of the 34 dyads in the deep condition reported lower-than-average depth. When participants were mean-split into high versus low self-reported depth groups, those in the high-depth group reported significantly greater feelings of connection (*M* = 3.54) than those in the low-depth group (*M* = 3.12) (*t*(68) = 4.21, *P* < .001). Additionally, self-reported depth significantly predicted self-reported connection after the conversation (*β*  =  0.35, *t*(68) = 5.20, *P* < .0001).

To further probe the relationship between depth and connection in the reverse direction, we also mean-split dyads based on self-reported connection and used the felt depth variable as the dependent variable. High-connection dyads rated their conversations as significantly deeper (*M* = 2.86) than low-connection dyads (*M* = 2.33), (*t*(68) = 3.31, *P* = .002) (Fig. 4, middle). Together, these analyses indicate that both experimentally assigned and self-reported depth relate to subjective feelings of connection in conversation.

### Inter-brain synchrony and connection

**Figure 5:**
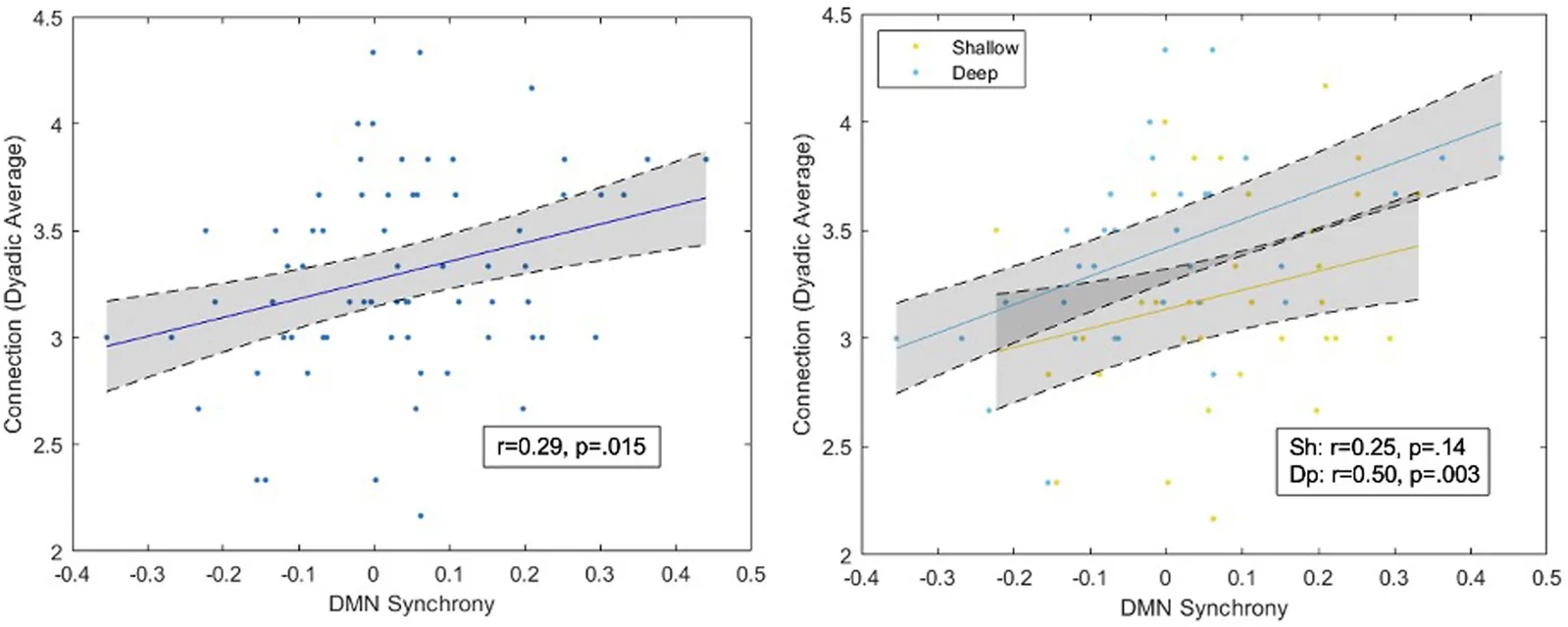
Correlations between neural synchrony in the DMN and key dependent variable connection. Left: DMN synchrony is positively correlated with self-reported connection. Right: The correlation between DMN synchrony and connection is stronger in the deep condition compared to the shallow condition.

We used linear regression to examine the relationship between dyadic inter-brain synchrony in each of the four neural networks (DMN, FPN, VAN, DAN) and self-reported connection in dyads. As hypothesized *a priori*, DMN inter-brain synchrony was significantly associated with self-reported connection after the conversation (β = 0.874, *t*(67) = 2.51, *P* = .015; see Fig. 5, left). Participants who reported high connection also exhibited significantly greater DMN synchrony (M = 0.071) than those who reported low connection (*M* = -0.004) (*t*(67) = 2.03, *P* = .047) (Fig. 4, right). In contrast, inter-brain synchrony in other networks did not significantly relate to connection, including FPN (β = 0.546, *t*(67) = 1.40, *P* = .166), VAN (β = 0.614, *t*(68) = 1.52, *P* = .134), and DAN (β = 0.785, *t*(47) = 1.13, *P* = .264). Because inter-brain synchrony in networks other than the DMN did not correlate with connection, the rest of our analyses focused on the DMN and its components.

As a control analysis, we generated pseudo-dyads by randomly pairing participants within the same condition who did not interact, and compared DMN synchrony between real and pseudo-dyads. DMN synchrony was significantly higher in real dyads relative to pseudo-dyads (*β*  =  0.046, *t*(137) = 2.20, *P* = .030). Together, these findings suggest that the association between DMN inter-brain synchrony and felt connection is specific to real interacting dyads and is unlikely to be explained by spurious synchrony arising from shared task structure alone.

To further understand the brain responses within the DMN, we conducted analyses on each of the DMN subregions, including mPFC and both left and right TPJ. Inter-brain synchrony in mPFC was significantly associated with self-reported connection (β = 0.900, *t*(68) = 2.42, *P* = .018). Inter-brain synchrony in right TPJ was the strongest predictor of self-reported connection (β = 1.485, *t*(64) = 3.12, *P* = .003) of any neural metric we examined, whereas inter-brain synchrony in left TPJ did not significantly relate to self-reported connection (β = 0.759, *t*(66) = 1.66, *P* = .102).

### Inter-brain synchrony, conversational depth, and connection

Because we had two measures of conversational depth (assigned and self-reported), we conducted separate regression analyses using each measure, along with DMN inter-brain synchrony, to examine their association with self-reported connection.

A regression model including assigned depth condition and DMN synchrony explained a significant proportion of variance in dyadic connection ($R_{\text{adjusted}}^{2}$ = 0.158, *F*(2,66) = 7.39, *P* = .001). Within this model, both depth condition (β = 0.300, *t*(66) = 2.80, *P* = .007) and DMN synchrony (β = 1.134, *t*(66) = 3.29, *P* = .002) contributed significantly to explaining self-reported connection.

A regression model examining self-reported depth and DMN synchrony as predictors of connection regardless of assigned condition also showed that the model significantly explains variance in connection ($R_{\text{adjusted}}^{2}$ = 0.368, *F*(2,66) = 20.8, *P* < .0001). Within this model, self-reported depth (β = 0.357, *t*(66) = 5.69, *P* < .0001) and DMN synchrony (β = 0.973, *t*(66) = 3.38, *P* = .001) both explained a significant amount of variance for self-reported connection. In the smaller subsamples of the shallow (*n* = 36) and deep (*n* = 34) dyads, DMN synchrony significantly correlated with self-reported connection in the deep condition (*r *= 0.50, *P* = .003), but not in the shallow condition (*r *= 0.25, *P* = .14) (Fig. 5, right).

To disentangle the unique contributions of DMN inter-brain synchrony and conversational depth in shaping connection, we examined the relationship between DMN synchrony and conversational depth. DMN inter-brain synchrony was significantly associated with depth condition as revealed by a logistic regression (β = -3.76, *z* = -2.15, *P* = .031) but in the opposite direction of what we expected, such that greater inter-brain synchrony was present in the shallow condition than the deep condition. When we looked at the self-reported depth, results were non-significant but showed the same unexpected direction of effect (β = -0.28, *t*(67) = -0.49, *P* = .623).

### Machine learning classification with inter-brain synchrony

To examine whether neural synchrony could be used to classify dyads self-reporting low or high connection, we next conducted a machine learning classification analysis using logistic regression with five-fold cross-validation. First, DMN inter-brain synchrony and depth condition assignment were used as features to predict self-reported connection. We observed accurate classifications of 60.2% dyads across 1,000 repetitions (*P* = .07). Then, DMN inter-brain synchrony and self-reported depth were used as features to predict self-reported connection. We observed accurate classifications of 64.5% across 1,000 repetitions (*P *= .012). When DMN inter-brain synchrony alone was used as the feature to predict self-reported connection, we observed accurate classifications of 55.0% across 1,000 repetitions (*P* = .19).

Using separate inter-brain synchrony estimates from DMN subregions (mPFC, left TPJ, and right TPJ) as features to predict self-reported connection, we observed accurate classifications of 61.4% across 1000 repetitions (*P* = .04). When mPFC, left TPJ, and right TPJ inter-brain synchrony features were each used alone to predict self-reported connection, we observed accurate classifications of 53.3% in mPFC (*P* = .36), 48.6% in left TPJ (*P* = .54), but 62.6% in right TPJ (*P* = .01).

## Discussion

Social neuroscience has made great strides in understanding how our social cognition is instantiated in patterns of brain activity, yet much of this work has examined individuals in isolation—lying in MRI scanners, observing social stimuli, or imagining interpersonal experiences. With the growing adoption of ecologically valid methods such as face-to-face hyperscanning with fNIRS, researchers can now capture real-time brain activity during actual social interactions, offering a window into the dynamic and reciprocal nature of human connection. Previous research has shown that individuals with strong pre-existing social bonds tend to exhibit greater DMN synchrony when passively viewing video clips (Parkinson *et al.* 2018; Li *et al.* 2022). However, this neural response pattern had not been examined as strangers form connections through face-to-face conversation.

Here, we provide the first evidence linking inter-brain synchrony during a live “get-to-know-you” conversation between strangers to subsequent self-reported feelings of interpersonal connection. Synchrony within the right TPJ subregion of the DMN emerged as the strongest individual neural predictor of self-reported connection across both regression analyses and machine learning classification. In parallel, both experimentally manipulated and self-reported measures of conversational depth predicted self-reported connection, replicating prior behavioral findings (Kardas *et al.* 2022). At a network level, DMN synchrony correlated with self-reported connection, and when combined with self-reported depth (a variable not correlated with DMN synchrony), machine learning produced significant classification accuracy of self-reported connection (64.5%). This pattern effect was observed in DMN, but not in the other networks we examined (FPN, DAN, VAN).

Unexpectedly, DMN synchrony did not predict self-reported depth. A possible explanation is that DMN synchrony, particularly in TPJ, reflects a form of shared social-cognitive alignment, an experience of “seeing eye-to-eye” and may contribute to a burgeoning connection without the conversation feeling deeper. In contrast, self-reported depth may reflect more deliberate appraisals about the conversation’s meaningfulness or emotional intimacy. From this perspective, while DMN synchrony may indicate pre-reflective alignment during the interaction, perceived depth may emerge from more effortful post hoc evaluations. At the same time, an alternative interpretation is plausible: deeper conversations may recruit more self-referential and affectively elaborative processing, both of which have been linked to default-mode-network function (Maillet *et al.* 2019). Under such conditions, participants may become more inwardly focused and relatively less attuned to their partner’s ongoing cues in the moment. On this account, the absence of a positive relationship between depth and DMN inter-brain synchrony is not necessarily surprising. Together, these findings suggest that social connection may relate to both automatic neural alignment and conscious cognitive reflection—two complementary but distinct pathways.

A related consideration is that DMN inter-brain synchrony and self-reported depth operate on different temporal scales. Self-reported depth is a retrospective summary judgment of the entire 20-min conversation and, like other retrospective evaluations of affective episodes, may be disproportionately weighted by emotional peaks and endpoints (i.e. the “peak-end rule”) (Fredrickson and Kahneman 1993). DMN inter-brain synchrony, in contrast, is computed from moment-to-moment brain activity and is therefore better positioned to track interpersonal alignment as it unfolds in real time. Combining continuous self-report measures (e.g. dial ratings of perceived connection during the conversation) with dynamic synchrony estimates in future studies could help disentangle these momentary versus retrospective contributions to felt connection.

A related possibility is that inter-brain synchrony itself might come in qualitatively different varieties, only some of which might relate to the psychological connection between two people who “see the world” in the same way. For instance, there might be a difference between “easy” and “hard” inter-brain synchrony. Easy synchrony may occur when people engage in the same movements (Marton-Alper *et al.* 2023). In a conversational context, such easy synchrony might occur during shallow conversations when discussing simple events for which both members of the dyad have strong familiarity. If a student describes walking along a path on campus to another student from the same campus, the listener could easily follow along and produce synchrony despite the lack of depth or relevance to later feelings of connection.

In contrast, hard synchrony would involve effortful co-construction of meaning during emotionally or cognitively complex conversations. Sharing a painful memory or revealing a personal truth requires more engagement and mutual attunement to reach alignment. Reaching inter-brain synchrony from such conversations would then reflect mutual understanding and psychological connection. While intersubject correlation may not be able to distinguish these two varieties of synchrony, other methods, such as the off-diagonal delay in cross-recurrence quantification analysis (Goldstein *et al.* 2025), may be better suited to do so.

An important avenue for future research involves exploring methods to better pinpoint the precise moments when the connection between two people is growing or fading. Countless articles and blog posts make pronouncements about the best ways to make friends, but little is known about the key moments in first conversations that are critical to moving people toward or away from a closer connection. Identifying such temporal dynamics through neuroimaging would allow researchers to trace back the corresponding verbal and nonverbal cues, thereby advancing our understanding of the processes that facilitate or inhibit connection formation.

A metric like inter-brain synchrony that might reflect conversation-critical social cognitive processes and that does not interfere with the flow of the conversation might be a solution. In addition, the recently developed Dynamic Interactions and Multimodal Signals (DIMS) Dashboard enables researchers to combine quantitative metrics with qualitative behavioral markers captured in video recordings of the interaction (Miao *et al.* 2025). Using this tool, researchers can visually identify noticeable features of a signal (e.g. rises over several seconds) to qualitatively examine the corresponding segment of the conversation. By linking neural or behavioral fluctuations with observable cues such as verbal repetition or head nods, this qualitative-quantitative integration grounds computational findings in lived behavior and renders theoretical constructs like “connection” more interpretable.

More broadly, the current work may inform interventions aimed at fostering deeper connections in everyday life. Prior work shows that people often make inaccurate predictions about the outcomes of talking to strangers: underestimating how enjoyable and meaningful such interactions can be while overestimating their potential awkwardness (Boothby *et al.* 2018; Kardas *et al.* 2022). The use of inter-brain synchrony as a continuous measure of shared engagement offers a way to assess whether the interaction itself is changing (whether two people are coming into or out of alignment) rather than relying solely on post-conversation reports. Such dynamic measurement could eventually inform interventions that target the process of connection as it unfolds, rather than its aftermath, helping to identify moments when conversational depth and mutual understanding are actively developing.

Future work should also examine other key factors like participants’ traits and state features, such as personality or current level of loneliness. There are also open questions about the extent to which conversation-based synchrony within DMN is a marker of seeing eye-to-eye in other contexts, such as cross-ideological conversations, negotiations, classroom education, therapy, performance within teams, and even interactions with AI agents. Future work is needed to determine the breadth and limits of application of fNIRS-based synchrony during social interactions. Additionally, an important limitation of the present work concerns our framing of DMN inter-brain synchrony as an index of “seeing eye-to-eye.” Prior fMRI work has linked greater DMN synchrony to similarity in subjective interpretations and shared understanding during passive viewing of naturalistic stimuli (Yeshurun *et al.* 2017; Nguyen *et al.* 2019; Saalasti *et al.* 2019; Dieffenbach *et al.* 2021; Lieberman 2025). Indeed, activity in right TPJ is the region of the brain most strongly associated with shared ways of experiencing stories (Lieberman 2025). We adopt this prior evidence as our premise here, but the present study did not directly assess the extent to which dyads shared interpretations or felt they “saw eye-to-eye” during the conversation. Our primary behavioral outcome was felt interpersonal connection, which is conceptually distinct from common ground or similarity of beliefs: two people can feel deeply connected while still disagreeing on opinions, values, or interpretations. Future work could test the eye-to-eye premise more directly.

Before finishing, we should consider the ways that our results relate to the study most similar to ours (Speer *et al.* 2024). At first glance, our results may seem to be at odds with those of Speer *et al.* They found that friends showed greater divergence in neural state space than strangers as they spoke to one another in fMRI scanners. While inter-brain synchrony and movement through neural state space are distinct measures, they are not orthogonal; perfect synchrony throughout the brain would necessitate convergence in neural state space as well. It is important to note that the differences between strangers and friends in their study may reflect pre-existing differences in these groups in addition to within-task dynamics. While we compared strangers who came to feel connected to those who did not, Speer *et al.* compared dyads who entered the study already connected or not. It may be the case that the dynamics that predict successful conversations among friends are different from those corresponding to strangers coming to feel more connected. Additionally, Speer *et al.* found that even among strangers, those who diverged more in neural state space enjoyed the conversation more, but this did not translate to greater feelings of closeness, which would be most analogous to our measure of connection. Finally, it is of note that Speer *et al.* were examining dyads lying in separate MRI scanners, whereas our dyads were sitting face-to-face at a table, able to show and detect a wide range of nonverbal behaviors that may have served to both synchronize neural activity and drive feelings of connection. That said, both studies show important relationships between the similarities and differences in neural activity while people have conversations with one another and suggest more work is needed in this space.

In conclusion, this study provides the first direct evidence that inter-brain synchrony within the default mode network, particularly in the right TPJ, predicts the formation of interpersonal connections during naturalistic face-to-face conversations between strangers. This suggests that DMN coupling may serve as a neural marker of emerging social connection. Using fNIRS hyperscanning and machine learning, we demonstrated that DMN synchrony, in conjunction with perceived conversational depth, reliably distinguished high- from low-connection dyads. These findings extend prior work on DMN synchrony during passive co-viewing to real-time, face-to-face interactions, supporting the hypothesis that DMN synchrony functions as a neural index of “seeing eye-to-eye.”

## Data Availability

The general fNIRS pre-processing pipeline can be accessed at https://github.com/abinnquist/fNIRSpreProcessing. Data and code specific to this article can be accessed at https://github.com/miaoqy0729/ConnectionStrangerDMN.
